# Personalized treatment decision algorithms for the clinical application of serum neurofilament light chain in multiple sclerosis: A modified Delphi Study

**DOI:** 10.1177/13524585251335466

**Published:** 2025-04-28

**Authors:** Özgür Yaldizli, Pascal Benkert, Lutz Achtnichts, Amit Bar-Or, Viviane Bohner-Lang, Claire Bridel, Manuel Comabella, Oliver Findling, Giulio Disanto, Sebastian Finkener, Claudio Gobbi, Cristina Granziera, Marina Herwerth, Robert Hoepner, Dana Horakova, Nicole Kamber, Michael Khalil, Philipp Kunz, Patrice Lalive, Ralf Linker, Johannes Lorscheider, Stefanie Müller, Johanna Oechtering, Victoria Pettypool, Fredrik Piehl, Caroline Pot, Patrick Roth, Marie Théaudin, Mar Tintore, Carmen Tur, Denis Uffer, Marjolaine Uginet, Jochen Vehoff, Heinz Wiendl, Tjalf Ziemssen, Chiara Zecca, Anke Salmen, David Leppert, Tobias Derfuss, Ludwig Kappos, Lars G Hemkens, Perrine Janiaud, Jens Kuhle

**Affiliations:** Department of Clinical Research, University Hospital Basel, University of Basel, Basel, Switzerland; Research Center for Clinical Neuroimmunology and Neuroscience Basel (RC2NB), University Hospital Basel, University of Basel, Basel, Switzerland; Neurologic Clinic and Policlinic, MS Centre, University Hospital Basel, Basel, Switzerland; Translational Imaging in Neurology Basel, Department of Medicine and Biomedical Engineering, University Hospital Basel, University of Basel, Basel, Switzerland; Department of Clinical Research, University Hospital Basel, University of Basel, Basel, Switzerland; Neurozentrum Oberaargau, Langenthal, Switzerland; Center for Neuroinflammation and Experimental Therapeutics and Department of Neurology, Perelman School of Medicine, University of Pennsylvania, Philadelphia, PA, USA; Patient Consultant, Basel, Switzerland; Translational Biomarker Research Group, Department of Medicine, Faculty of Medicine, University of Geneva, Geneva, Switzerland; Multiple Sclerosis Centre of Catalonia (Cemcat), Vall d’Hebron Barcelona Hospital, Barcelona, Spain; Department of Neurology, Cantonal Hospital Aarau, Aarau, Switzerland; Multiple Sclerosis Center, Neurocenter of Southern Switzerland, Ospedale Regionale di Lugano (EOC), Lugano, Switzerland; Department of Neurology, Cantonal Hospital Aarau, Aarau, Switzerland; Multiple Sclerosis Center, Neurocenter of Southern Switzerland, Ospedale Regionale di Lugano (EOC), Lugano, Switzerland; Research Center for Clinical Neuroimmunology and Neuroscience Basel (RC2NB), University Hospital Basel, University of Basel, Basel, Switzerland; Neurologic Clinic and Policlinic, MS Centre, University Hospital Basel, Basel, Switzerland; Translational Imaging in Neurology Basel, Department of Medicine and Biomedical Engineering, University Hospital Basel, University of Basel, Basel, Switzerland; Department of Neurology, University Hospital Zurich, Zurich, Switzerland; Institute of Pharmacology and Toxicology, University of Zurich, Zurich, Switzerland; Department of Neurology, Klinikum rechts der Isar, Technical University of Munich, Munich, Germany; Department of Neurology, Bern University Hospital, University of Bern, Bern, Switzerland; Department of Neurology and Center of Clinical Neuroscience, First Faculty of Medicine, Charles University in Prague and General University Hospital, Prague, Czech Republic; Division of Neurology, Department of Internal Medicine, Bürgerspital Solothurn, Solothurn, Switzerland; Department of Neurology, Medical University of Graz, Graz, Austria; Patient Consultant, Basel, Switzerland; Division of Neurology, Department of Clinical Neurosciences, Faculty of Medicine, Geneva University Hospitals, Geneva, Switzerland; Department of Neurology, University of Regensburg, Regensburg, Germany; Research Center for Clinical Neuroimmunology and Neuroscience Basel (RC2NB), University Hospital Basel, University of Basel, Basel, Switzerland; Neurologic Clinic and Policlinic, MS Centre, University Hospital Basel, Basel, Switzerland; Department of Neurology, University Teaching and Research Hospital St. Gallen, St. Gallen, Switzerland; Research Center for Clinical Neuroimmunology and Neuroscience Basel (RC2NB), University Hospital Basel, University of Basel, Basel, Switzerland; Neurologic Clinic and Policlinic, MS Centre, University Hospital Basel, Basel, Switzerland; Patient Consultant, Basel, Switzerland; Department of Neurology, Karolinska University Hospital, Stockholm, Sweden; Neuroimmunology Unit, Center for Molecular Medicine, Karolinska Institute, Stockholm, Sweden; Service of Neurology, Department of Clinical Neurosciences, Lausanne University Hospital (CHUV), University of Lausanne, Lausanne, Switzerland; Department of Neurology, University Hospital Zurich, University of Zurich, Zurich, Switzerland; Service of Neurology, Department of Clinical Neurosciences, Lausanne University Hospital (CHUV), University of Lausanne, Lausanne, Switzerland; Multiple Sclerosis Centre of Catalonia (Cemcat), Vall d’Hebron Barcelona Hospital, Barcelona, Spain; Multiple Sclerosis Centre of Catalonia (Cemcat), Vall d’Hebron Barcelona Hospital, Barcelona, Spain; Department of Neurology, University Teaching and Research Hospital St. Gallen, St. Gallen, Switzerland; Division of Neurology, Department of Clinical Neurosciences, Faculty of Medicine, Geneva University Hospitals, Geneva, Switzerland; Department of Neurology, University Teaching and Research Hospital St. Gallen, St. Gallen, Switzerland; Department of Neurology, Institute of Translational Neurology, University Hospital Münster, Münster, Germany; Center of Clinical Neuroscience, Neurological Clinic, University Hospital Carl Gustav Carus, TU Dresden, Dresden, Germany; Multiple Sclerosis Center, Neurocenter of Southern Switzerland, Ospedale Regionale di Lugano (EOC), Lugano, Switzerland; Faculty of Biomedical Sciences, Università della Svizzera Italiana (USI), Lugano, Switzerland; Department of Neurology, Ruhr-University Bochum, St. Josef-Hospital, Bochum, Germany; Research Center for Clinical Neuroimmunology and Neuroscience Basel (RC2NB), University Hospital Basel, University of Basel, Basel, Switzerland; Research Center for Clinical Neuroimmunology and Neuroscience Basel (RC2NB), University Hospital Basel, University of Basel, Basel, Switzerland; Neurologic Clinic and Policlinic, MS Centre, University Hospital Basel, Basel, Switzerland; Research Center for Clinical Neuroimmunology and Neuroscience Basel (RC2NB), University Hospital Basel, University of Basel, Basel, Switzerland; Department of Clinical Research, University Hospital Basel, University of Basel, Basel, Switzerland; Research Center for Clinical Neuroimmunology and Neuroscience Basel (RC2NB), University Hospital Basel, University of Basel, Basel, Switzerland; Research Center for Clinical Neuroimmunology and Neuroscience Basel (RC2NB), University Hospital Basel, University of Basel, Basel, Switzerland; Research Center for Clinical Neuroimmunology and Neuroscience Basel (RC2NB), University Hospital Basel, University of Basel, Basel, Switzerland; Departments of Biomedicine and Clinical Research, University Hospital Basel, University of Basel, Basel, Switzerland; Department of Neurology, University Hospital Basel, University of Basel, Basel, Switzerland; Multiple Sclerosis Centre, University Hospital Basel, Basel, Switzerland

**Keywords:** personalized treatment strategies, serum neurofilament light chain, Delphi study, escalation, de-escalation

## Abstract

**Background:**

Serum neurofilament light (sNfL) chain levels, a sensitive measure of disease activity in multiple sclerosis (MS), are increasingly considered for individual therapy optimization yet without consensus on their use for clinical application.

**Objective:**

We here propose treatment decision algorithms incorporating sNfL levels to adapt disease-modifying therapies (DMTs).

**Methods:**

We conducted a modified Delphi study to reach consensus on algorithms using sNfL within typical clinical scenarios. sNfL levels were defined as “high” (>90th percentile) vs “normal” (<80th percentile), based on normative values of control persons. In three rounds, 10 international and 18 Swiss MS experts, and 3 patient consultants rated their agreement on treatment algorithms. Consensus thresholds were defined as moderate (50%–79%), broad (80%–94%), strong (≥95%), and full (100%).

**Results:**

The Delphi provided 9 escalation algorithms (e.g. initiating treatment based on high sNfL), 11 horizontal switch (e.g. switching natalizumab to another high-efficacy DMT based on high sNfL), and 3 de-escalation (e.g. stopping DMT or extending intervals in B-cell depleting therapies).

**Conclusion:**

The consensus reached on typical clinical scenarios provides the basis for using sNfL to inform treatment decisions in a randomized pragmatic trial, an important step to gather robust evidence for using sNfL to inform personalized treatment decisions in clinical practice.

## Introduction

Therapeutic strategies in multiple sclerosis (MS) aim to reduce the relapse rate and to slow disability accumulation. There are more than 20 disease-modifying therapies (DMTs) available, including high-efficacy drugs like natalizumab,^1^ alemtuzumab,^2^ and B-cell depleting therapies (BCDT: rituximab, ocrelizumab, ofatumumab, and ublituximab),^3–5^ which provide close to complete suppression of acute inflammatory disease activity. However, such DMTs interfere with physiological immune response functions putting persons with (pw) MS at risk of infections.^6^

Several studies have demonstrated the benefits of early therapy initiation or early escalation to high-efficacy DMTs (HET) in relapsing-remitting (RR) MS (“hit hard, hit early” paradigm).^6,7^ However, controversies persist regarding the pursue of such aggressive strategy due to potential longer-term side effects.^8^

Recent smaller studies have also shown that de-escalation could be a safe option in RRMS, decreasing the exposure to MS therapies without recurrence of disease activity.^9–11^ Conversely, the observation that reduced drug exposure negatively impacts disease progression^12,13^ suggests that a similar negative effect may occur when de-escalating the dose. Currently, there is no clear consensus on how or when to de-escalate, creating uncertainties and making shared-decision making between physician and patient challenging. Hence, a more personalized or precision medicine approach to MS therapy is urgently needed.^14,15^

A promising tool is the use of neurofilament light chain (NfL)^16,17^ as an additive criterion. NfL is a marker of neuroaxonal damage^16^ that can be measured in a standardized way in blood making it suitable for use in clinical practice. Serum NfL (sNfL) has been demonstrated as a useful biomarker for both acute inflammatory disease activity and disability accumulation, and treatment response, in real-world settings^18–21^ and randomized controlled trials.^22,23^ However, the practical use and added value of this biomarker as part of individual therapeutic decision-making has not been evaluated so far.^24^

As part of an ongoing pragmatic randomized trial assessing the added value of informing treatment decisions by 6-monthly sNfL monitoring on patient relevant outcomes (MultiSCRIPT trial—NCT06095271),^25^ we conducted this Delphi study. The purpose was to reach a consensus among experts in the field closely involved with the trial and patient consultants on treatment decision algorithms on the inclusion of sNfL information to guide individual patient level treatment decisions for common clinical scenarios encountered in usual care.

## Materials and methods

We conducted a modified Delphi study^26^ in three rounds (two online and one in-person) over a period of 3 months (26 October 2022 till 27 January 2023). The Delphi methodology aims to gather experts’ opinions until a consensus is reached on a topic—here treatment decision algorithms—through an iterated, structured voting process. The multiple survey rounds allow participants to nuance and reconsider their decisions based on the anonymized votes and comments in the previous online rounds, followed by a moderated discussion during the final in-person round.^26,27^

### Definition: NfL levels

Serum NfL levels are age- and body mass index (BMI)-dependent, thus their normalized values (i.e. adjusted sNfL percentiles or Z scores based on healthy controls) are more meaningful for the clinical interpretation in individual pwMS and to prognosticate future clinical disease activity^20,28–30^ than the absolute sNfL concentration. Percentiles/Z scores are interchangeable and reflect the deviation of a pwMS’s sNfL from the mean value of age- and BMI-matched healthy individuals (50th percentile; Z score 0). sNfL percentiles/Z scores identify a gradually increased risk of future acute inflammatory events (relapse, lesion formation) as well as worsening of disability.^20^ We defined “high” sNfL as >90th percentile.^20^ This is based on evidence indicating that pwMS with levels higher than the 90th percentile (Z score 1.28) have about a two-fold higher risk for any sign of clinical or disease activity on magnetic resonance imaging (MRI) in the following year (RR 2.28; 95% confidence interval (CI): 1.11–4.68; *p* = 0.025).^20^ Before applying the treatment decision algorithms based on sNfL values, other possible causes of high sNfL need to be considered such as, for example, head trauma, stroke, relevant sports-related head injury, at least medium severe renal failure (glomerular filtration ratio (GFR) < 60 mL/min/1.73 m^2^), suboptimal treated diabetes mellitus or any other concomitant disease including incipient neurodegenerative diseases that may lead to relevant neuro-axonal damage.

Serum NfL values <80th percentile are values typically seen in healthy control subjects and were considered as “normal” sNfL in comparison with high sNfL.

### Definition: no evidence of disease activity

No evidence of disease activity (NEDA) since last visit was defined as NEDA2 with no relapses and no Expanded Disability Status Scale (EDSS) worsening or as NEDA3 with additionally no MRI activity.

### Definition: low, medium and HET

During round 1, participants were asked to classify all DMTs commonly used in Switzerland (i.e. glatiramer acetate, interferon-beta formulations, teriflunomide, dimethyl fumarate, S1P receptor modulators, cladribine, alemtuzumab, BCDT (rituximab, ocrelizumab, ofatumumab), natalizumab) into low, medium, and high efficacy. Based on round 1, the final classification was then constructed and voted on during the second round.

### Algorithms development

The core team (ÖY, PB, CZ, AS, DL, TD, LK, LGH and JK) identified two main settings (1) escalation or horizontal treatment change in case high sNfL and (2) de-escalation in case sNfL <80th percentile. Depending on the currently used DMT (i.e. low, medium or high-efficacy), treatment decision algorithms were further grouped into topics (e.g. escalation from low to medium or HET).

### Panel selection

Before the first round of the Delphi study, we invited local investigators (all attending MS neurologists) from the 8 Swiss MS Cohort (SMSC) centers taking part in the MultiSCRIPT trial, international senior MS experts, and patient consultants.

### Consensus

For the two online surveys, participants were asked to rate their agreement with the different treatment decision algorithms using a 9-Likert-type scale (1 *Strongly disagree* to 9 *Strongly agree*). For the last in-person round, participants were asked to vote by show of hands “agree” or “disagree.” The following agreement thresholds were used: <50% excluded, 50%–79% moderate consensus, 80%–94% broad consensus, 95%–99% strong consensus, and 100% full agreement. More information on the voting rounds in Supplemental Material.

## Results

**Figure 1.**
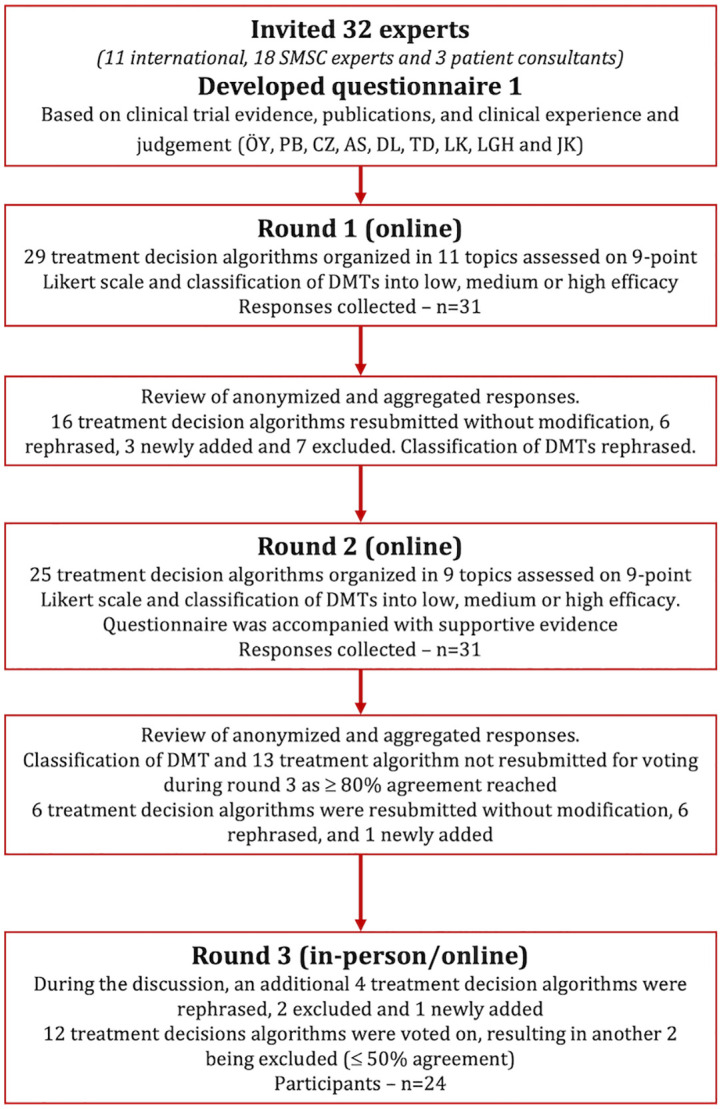
Flow chart of the Delphi process.

Thirty-two participants were invited and accepted to participate, of which, 18 were experts from the SMSC centers (Basel, Bern, Zurich, Lugano, St Gallen, Aarau, Geneva and Lausanne), 11 international senior MS experts (Germany (*n* = 4), Spain (*n* = 3), and United States, Czech Republic, Sweden and Austria (*n* = 1 each)), and 3 patient consultants. All, except one, participated in both online rounds and 24 participants were present at the hybrid open discussion meeting (14 experts from the SMSC centers, 9 international experts and 1 patient consultant). Of note, all SMSC centers were represented in round 3. Figure 1 provides a detailed overview of the modified Delphi Study. Surveys for rounds 1 and 2 are available as Supplemental Material.

### Classification of DMTs

A full agreement (100%) was reached for HET to include alemtuzumab, BCDT and natalizumab, a strong consensus (97%) for low efficacy DMTs to include glatiramer acetate, interferon beta and teriflunomide, and a broad consensus (83%) for medium efficacy DMTs to include dimethyl fumarate, S1P receptor modulators and cladribine.

### High NfL: escalation algorithms

#### From untreated to initiating DMT

**Figure 2.**
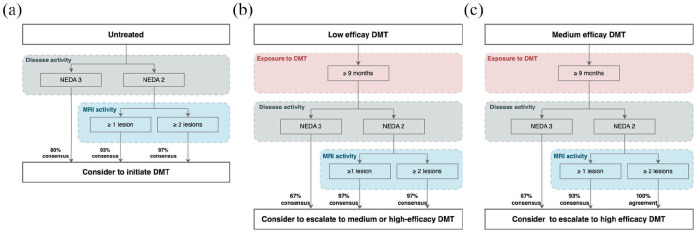
Escalation algorithms in patients with high sNfL (>90th percentile): (a) From untreated to initiating DMT; (b) From low to medium or high efficacy DMT; and (c) From medium to high efficacy DMT. Example of algorithm: If your patient is currently receiving a low efficacy DMT for at least 9 months and has high sNfL (>90th percentile) jointly consider with your patient to escalate to medium or HET if your patient has NEDA2 plus MRI activity with at least 1 unequivocal new/enlarging T2w lesion or contrast enhancing T1w lesion. DMT: Disease Modifying Therapy; MRI: magnetic resonance imaging; NEDA: No evidence of disease activity was defined as NEDA2 i.e. no relapses and no Expanded Disability Status Scale (EDSS) worsening or as NEDA3 no relapses, no EDSS worsening and no MRI activity.

For pwMS with NEDA3 who are untreated but have high sNfL, we reached broad consensus (80% agreement) to initiate a DMT (Figure 2(a)); agreement was stronger when there was additionally MRI activity (93%–97%).

#### From low to medium or HET

For pwMS with NEDA3 and high sNfL, moderate consensus was reached (67%) to escalate from low efficacy DMT (Figure 2(b)) to a medium or HET. Experts emphasized that pwMS needed to be on the low efficacy DMT for at least 9 months to exclude high sNfL levels due to the DMT not yet having reached full efficacy; the agreement was stronger when pwMS also showed MRI activity (97%).

#### From medium to HET

In NEDA3 with high sNfL, moderate consensus was reached (67%) to escalate from a medium to HET (Figure 2(c)). Agreement was again stronger with MRI activity: broad consensus (93%) with one new/enlarging lesion and a full agreement (100%) with at least two new/enlarging lesions.

### High NFL: horizontal switch algorithms

#### Switching from natalizumab

**Figure 3.**
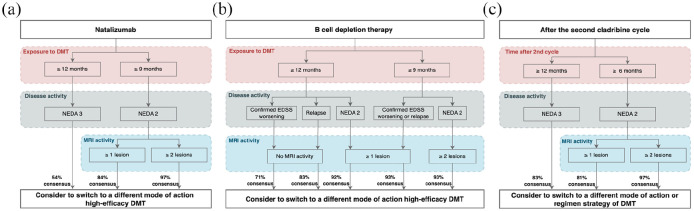
Horizontal switch algorithms in patients with high sNfL (>90th percentile): (a) From natalizumab; (b) From B-cell depletion therapy; and (c) From cladribine to different mode of action of DMT. Example of algorithm: If your patient is currently receiving B-cell depletion therapy for at least 12 months and has high sNfL (>90th percentile) jointly consider with your patient to switch to a different mode of action HET if your patient has relapses but no MRI activity. EDSS: Expanded Disability Status Scale; DMT: Disease-Modifying Therapy; MRI: magnetic resonance imaging; NEDA: No evidence of disease activity was defined as NEDA2, i.e., no relapses and no EDSS worsening or as NEDA3 no relapses, no EDSS worsening and no MRI activity.

During round 1, consensus on treatment algorithms to shorten natalizumab (Figure 3(a)) intervals from 6 to 4 weeks were divided with 31% disagreeing, 38% undecided, and 31% agreeing to shorten treatment intervals for pwMS with NEDA3 and high sNfL. The algorithms were dropped (Supplemental Material).

Experts were reluctant (37% agreement) to switch natalizumab treatment after 9 months to another HET in pwMS with NEDA3 and only high sNfL. However, with a minimum treatment duration to 12 months, 54% agreed on switching from natalizumab to another HET with a different mode of action.

Agreement increased if there were additional signs of disease, for example, MRI activity; most experts would then switch even if the treatment duration was only 9 months with 84%–97% agreement depending on the number of new/enlarging lesions (one or two and more lesions).

#### Switching from BCDT

Experts were reluctant (20% in agreement, 30% undecided and 50% in disagreement) to switch from BCDT (Figure 3(b)) in pwMS with NEDA3 and high sNfL. The treatment decision algorithms were dropped following round 1 (Supplemental Material).

However, if pwMS on BCDT with high sNfL showed clinical and/or MRI activity, most experts would switch to another HET with a different mode of action. Consensus ranged from moderate (71%) to broad (93%) depending on the additional sign of disease activity and depending on the duration (i.e. ⩾9 or 12 months) of the BCDT.

#### Switching from cladribine

There was a broad consensus (83%) to switch from cladribine (Figure 3(c)) to another DMT if pwMS with NEDA3 showed high sNfL. However, experts recommended to switch only if high sNfL occurs 12 months after the second cycle, allowing the medication to exert its full effect. If pwMS on cladribine have both high sNfL and MRI activities, agreements ranged from 81% to 97% depending on the number of new/enlarging lesions to switch cladribine to a drug with a different mode of action even 6 months after the second cycle.

### sNfL < 80th percentile: De-escalation algorithm

#### From HET

During round 1, de-escalation algorithms for extending natalizumab treatment intervals from 4 to 6 weeks reached 72% agreement. However, it was dropped as it is already according to standards. Most experts were not in favor of stopping (10% agreement) or de-escalating (21%) BCDT to a lower efficacy DMT pwMS with NEDA3 who have sNfL <80th percentile. Those algorithms were dropped after round 1 (Supplemental Material).

In the first two rounds, de-escalation based on B-cell counts (i.e. extending treatment interval as long as B-cells are fully depleted) was introduced but dropped in round 3 due to lack of evidence on safety and efficacy of such an approach.^31^

**Figure 4.**
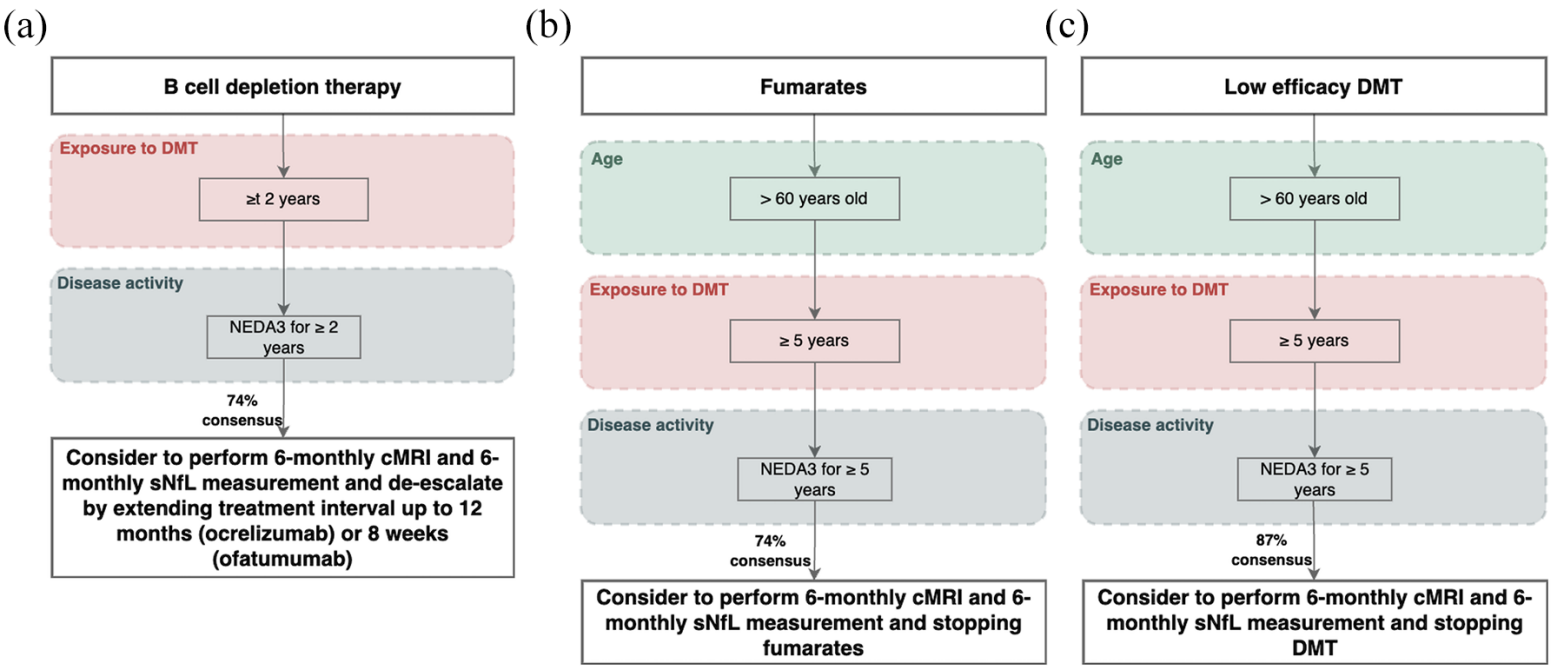
De-escalation algorithms in patients with “normal” sNfL (<80th percentile): (a) From B-cell depletion therapy to extending treatment interval; (b) From fumarates to stopping fumarates; and (c) From low efficacy DMT to stopping DMT treatment. Example of algorithm: If your patient wishes to stop treatment and is >60 years old, currently receiving low efficacy DMT for at least 5 years, has NEDA3 for the past 5 years and has normal sNfL (<80th percentile) jointly consider with your patient to perform 6-monthly cMRI and 6-monthly sNfL measurement and stopping DMT treatment. DMT: disease-modifying therapy; MRI: magnetic resonance imaging; NEDA: no evidence of disease activity was defined as NEDA3 no relapses, no EDSS worsening and no MRI activity.

De-escalation was recommended for pwMS on BCDT when they are NEDA3 for at least 2 years and have sNfL <80th percentile (Figure 4(a)), with a moderate consensus (74%) to extend the treatment interval up to 12 months for ocrelizumab and rituximab, or 8 weeks for ofatumumab.

#### From medium efficacy DMTs

Panelists highlighted the need to differentiate between S1P modulators and dimethyl fumarates due to concerns regarding the potential rebound of severe disease activity after stopping S1P modulators,^32^ which is not known to occur with dimethyl fumarate (Figure 4(b)).

De-escalation to a lower efficacy DMT from S1P receptor modulators or dimethyl fumarates were dropped due to insufficient agreement (below 50%; 26% and 48% respectively). A moderate consensus (74%) was reached to stop dimethyl fumarates in pwMS with sNfL <80th percentile and NEDA3 for the past 5 years when introducing an age limit and a minimum treatment duration: above 60 years of age and receiving fumarates for at least 5 years.

#### From low efficacy DMT

Similarly, a broad consensus (87% t) was reached to stop low efficacy DMT in pwMS over 60 years old, treated for at least 5 years, and with NEDA3 for the past 5 years and sNfL <80th percentile.

For monitoring and safety reasons, for all de-escalation algorithms, it was advised to perform 6-monthly cMRI and sNfL measurements, and to re-escalate if re-occurrence of disease activity (clinically, by MRI or high sNfL).

## Discussion

This Delphi study resulted in a consensus on the classification of DMTs as low, medium and high-efficacy, 9 escalation treatment decision algorithms (7 ranging from broad (≥80% agreement) to full agreement (100%) and 2 with a moderate consensus (50% to 79% agreement)), 11 horizontal treatment changes decision algorithms (9 broad consensus (≥80% agreement) and 2 moderate consensus (50% to 79% agreement)), and 3 de-escalation treatment decision algorithms (1 broad consensus (≥80% agreement) and 2 moderate consensus (50% to 79% agreement)).

The rationale to escalate DMT based on sNfL values is derived from studies showing that higher efficacy drugs lead to lower sNfL values compared to pwMS without treatment or on lower efficacy drugs.^20,22,23^ It also has been shown that sNfL is increased in a proportion of pwMS with NEDA3, and significantly prognosticating disease activity in the following years illustrating that sNfL and MRI provide only partially overlapping information on the future disease course. Although consensus increases with additional imaging disease activity, sNfL does capture disease activity beyond conventional clinical and imaging measures^20^ and potentially capturing it earlier.^33^ MRI lacks sensitivity for lesions below detectability by imaging and spinal cord MRI are less frequently done in routine—sNfL could complement those gaps in detection. Importantly, data from the OPERA studies have shown that persons with RRMS on ocrelizumab with high sNfL at week 48 after treatment start had a significantly higher chance for future disability accumulation up to 9 years after starting the DMT.^34^

Patients’ preference was frequently highlighted along with the concern in the de-escalation questions, on the risk of changing a treatment that works and has achieved NEDA3. Although patients who de-escalate cannot do better in terms of relapse rate or EDSS worsening, de-escalation might increase the quality of life in these patients due to fewer infusions, less risk for side effects/risks (also long-term) and reduce treatment burden and costs.

The long-term effects of immunosuppressive treatments like BCDT are not fully known. The immune system is essential for combating infections and providing tumor cell surveillance, making it challenging to balance better-defined shorter-term outcomes against the less understood long-term (10–50 years) benefits and harms.

Another drawback with de-escalation pertains to progression independent of relapse activity (PIRA). De-escalation may lead to subsequent disease activity as subtle inflammation might be missed by NfL measurements, clinical assessment, or MRI at the time of the decision.

Our understanding of the extent to which DMTs contributes to the pure neurodegenerative aspect of the disease remains limited. For example, ocrelizumab has been showed to reduce disability accumulation in primary progressive (PP)MS (ORATORIO trial) suggesting that its mode of action exerts this by suppressing brain diffuse inflammation that remains below detection threshold by standard measures. By de-escalating DMT, we may expose patients to a risk for developing more disability accumulation in the longer term. On the contrary, the effect size of ocrelizumab on disability progression was at best moderate (24%) in the ORATORIO trial,^35^ however, which still may be relevant for pwMS. If we believe that anti-inflammatory type DMTs might have a relevant effect on disability accumulation, de-escalation cannot be recommended as a standard unless their effect on this hidden inflammation can be quantitated by novel biomarkers.

Experts were reluctant to switch horizontally within HET with NEDA3 and high sNfL, likely because HET is often the “final” treatment option. However, consensus increased when additional signs of clinical or imaging disease activity where included in the treatment decision algorithms—as was the case for all algorithms. sNfL application in usual care is in its infancy which may explain the hesitancy from experts to base decisions solely on sNfL. The MultiSCRIPT trial will provide essential evidence to further understand the role of sNfL in treatment decisions and to conduct exploratory analyses on its impact on patient outcomes.

With the Delphi study, we aimed to provide a minimal set of treatment decision algorithms that experts have agreed upon for the most frequent clinical scenarios to be applied within the MultiSCRIPT trial^25^ in patients with RRMS. The herein proposed treatment decision algorithms may not apply to other clinical context. Caution is needed when using them more broadly, especially when elevated sNfL may be due to other potential causes (e.g. head trauma, stroke, relevant sports-related head injury, at least medium severe renal failure (GFR < 60 mL/min/1.73 m^2^), suboptimal treated diabetes mellitus or potential other incipient neurodegenerative diseases). This is particularly relevant in the absence of clinical or imaging evidence of disease activity. If uncertainty exists regarding the cause of high sNfL, repeat measurements may be warranted. Furthermore, factors such as comorbidities, neutralizing antibodies, among others, which may lead to suboptimal treatment effect, need to be factored in. The suggested algorithms assume that such special conditions do not apply if they do, decisions should follow best clinical practices, which may include maintaining the current treatment. They are non-binding, and patients and treating physician can always overrule the recommendations made here in the Delphi process.

Our findings illustrate the interest in using sNfL as a biomarker for personalized treatment decisions. sNfL monitoring offers additional information that may support earlier escalation of DMTs and safer de-escalation but is not intended to replace clinical and neuroimaging assessments. The added value of sNfL in informing treatment decision needs yet to be established; the MultiSCRIPT trial aims to provide such needed evidence.

The Delphi process has several limitations.

First, anonymity was lost in the final in-person meeting, which may have impacted willingness to express deviating views. However, the open discussion between experts allowed clarifications and to efficiently fine tune some of the algorithms.

Second, the first-round survey was drafted based on the core team expertise without formal systematic review of the literature. Personalization of treatment strategies in MS is only at its infancy and the evidence regarding escalation and de-escalation strategies are currently limited. Still, it is unlikely that the core team was not aware of the research and trials done in the field.

Third but related, the panel was small with a majority Swiss MS experts. Performing the Delphi was motivated by the MultiSCRIPT trial^25^ conducted within the SMSC assessing the added value of informing treatment decisions by 6-monthly sNfL monitoring on patient relevant outcomes including NEDA3 and quality of life that started in February 2024.^36^ It was important to gather consensus among the physicians also taking part in the trial based on their expertise and knowledge of the evidence as in routine care. The treatment algorithms developed herein may be prone to changes as we learn from the evidence generated.

Fourth, there are no generally accepted threshold to define a consensus in a Delphi study. We chose to include all treatment decision algorithms that had reached ≥50% consensus acknowledging different thresholds of consensus (i.e. moderate < broad < strong < full agreement). The added value of sNfL to monitor disease activity in clinical practice remains to be determined. Those treatment decision algorithms are intended to help physicians and patients in the application of NfL in their decision-making within a clinical trial and to provide insight on what can be considered. They do not intend to be clinical guidelines nor to supplant patients’ and physicians’ preferences.

Finally, not all the experts in the panel could attend the hybrid meeting. One cannot exclude that the results reached in the three rounds are biased toward the experts present during the meeting. However, the majority was present (77%) and the bias was minimized as all members of the panel and core team are co-authors of the current manuscript.

## Conclusion

The treatment decision algorithms we have developed and agreed upon are steppingstones to implementing sNfL into clinical routine care toward a more personalized care for people with MS. The algorithms have recently been applied in a pragmatic randomized clinical trial embedded in the Swiss MS Cohort assessing the superiority of 6-monthly sNfL monitoring compared with usual care.^25^ The evidence generated during the trial will allow to adjust, if necessary, the treatment decision algorithms before being implemented in routine care.

## Supplemental Material

sj-docx-1-msj-10.1177_13524585251335466 – Supplemental material for Personalized treatment decision algorithms for the clinical application of serum neurofilament light chain in multiple sclerosis: A modified Delphi Study Supplemental material, sj-docx-1-msj-10.1177_13524585251335466 for Personalized treatment decision algorithms for the clinical application of serum neurofilament light chain in multiple sclerosis: A modified Delphi Study by Özgür Yaldizli, Pascal Benkert, Lutz Achtnichts, Amit Bar-Or, Viviane Bohner-Lang, Claire Bridel, Manuel Comabella, Oliver Findling, Giulio Disanto, Sebastian Finkener, Claudio Gobbi, Cristina Granziera, Marina Herwerth, Robert Hoepner, Dana Horakova, Nicole Kamber, Michael Khalil, Philipp Kunz, Patrice Lalive, Ralf Linker, Johannes Lorscheider, Stefanie Müller, Johanna Oechtering, Victoria Pettypool, Fredrik Piehl, Caroline Pot, Patrick Roth, Marie Théaudin, Mar Tintore, Carmen Tur, Denis Uffer, Marjolaine Uginet, Jochen Vehoff, Heinz Wiendl, Tjalf Ziemssen, Chiara Zecca, Anke Salmen, David Leppert, Tobias Derfuss, Ludwig Kappos, Lars G Hemkens, Perrine Janiaud and Jens Kuhle in Multiple Sclerosis Journal

sj-docx-2-msj-10.1177_13524585251335466 – Supplemental material for Personalized treatment decision algorithms for the clinical application of serum neurofilament light chain in multiple sclerosis: A modified Delphi Study Supplemental material, sj-docx-2-msj-10.1177_13524585251335466 for Personalized treatment decision algorithms for the clinical application of serum neurofilament light chain in multiple sclerosis: A modified Delphi Study by Özgür Yaldizli, Pascal Benkert, Lutz Achtnichts, Amit Bar-Or, Viviane Bohner-Lang, Claire Bridel, Manuel Comabella, Oliver Findling, Giulio Disanto, Sebastian Finkener, Claudio Gobbi, Cristina Granziera, Marina Herwerth, Robert Hoepner, Dana Horakova, Nicole Kamber, Michael Khalil, Philipp Kunz, Patrice Lalive, Ralf Linker, Johannes Lorscheider, Stefanie Müller, Johanna Oechtering, Victoria Pettypool, Fredrik Piehl, Caroline Pot, Patrick Roth, Marie Théaudin, Mar Tintore, Carmen Tur, Denis Uffer, Marjolaine Uginet, Jochen Vehoff, Heinz Wiendl, Tjalf Ziemssen, Chiara Zecca, Anke Salmen, David Leppert, Tobias Derfuss, Ludwig Kappos, Lars G Hemkens, Perrine Janiaud and Jens Kuhle in Multiple Sclerosis Journal

sj-docx-3-msj-10.1177_13524585251335466 – Supplemental material for Personalized treatment decision algorithms for the clinical application of serum neurofilament light chain in multiple sclerosis: A modified Delphi Study Supplemental material, sj-docx-3-msj-10.1177_13524585251335466 for Personalized treatment decision algorithms for the clinical application of serum neurofilament light chain in multiple sclerosis: A modified Delphi Study by Özgür Yaldizli, Pascal Benkert, Lutz Achtnichts, Amit Bar-Or, Viviane Bohner-Lang, Claire Bridel, Manuel Comabella, Oliver Findling, Giulio Disanto, Sebastian Finkener, Claudio Gobbi, Cristina Granziera, Marina Herwerth, Robert Hoepner, Dana Horakova, Nicole Kamber, Michael Khalil, Philipp Kunz, Patrice Lalive, Ralf Linker, Johannes Lorscheider, Stefanie Müller, Johanna Oechtering, Victoria Pettypool, Fredrik Piehl, Caroline Pot, Patrick Roth, Marie Théaudin, Mar Tintore, Carmen Tur, Denis Uffer, Marjolaine Uginet, Jochen Vehoff, Heinz Wiendl, Tjalf Ziemssen, Chiara Zecca, Anke Salmen, David Leppert, Tobias Derfuss, Ludwig Kappos, Lars G Hemkens, Perrine Janiaud and Jens Kuhle in Multiple Sclerosis Journal

sj-pdf-4-msj-10.1177_13524585251335466 – Supplemental material for Personalized treatment decision algorithms for the clinical application of serum neurofilament light chain in multiple sclerosis: A modified Delphi Study Supplemental material, sj-pdf-4-msj-10.1177_13524585251335466 for Personalized treatment decision algorithms for the clinical application of serum neurofilament light chain in multiple sclerosis: A modified Delphi Study by Özgür Yaldizli, Pascal Benkert, Lutz Achtnichts, Amit Bar-Or, Viviane Bohner-Lang, Claire Bridel, Manuel Comabella, Oliver Findling, Giulio Disanto, Sebastian Finkener, Claudio Gobbi, Cristina Granziera, Marina Herwerth, Robert Hoepner, Dana Horakova, Nicole Kamber, Michael Khalil, Philipp Kunz, Patrice Lalive, Ralf Linker, Johannes Lorscheider, Stefanie Müller, Johanna Oechtering, Victoria Pettypool, Fredrik Piehl, Caroline Pot, Patrick Roth, Marie Théaudin, Mar Tintore, Carmen Tur, Denis Uffer, Marjolaine Uginet, Jochen Vehoff, Heinz Wiendl, Tjalf Ziemssen, Chiara Zecca, Anke Salmen, David Leppert, Tobias Derfuss, Ludwig Kappos, Lars G Hemkens, Perrine Janiaud and Jens Kuhle in Multiple Sclerosis Journal

sj-pdf-5-msj-10.1177_13524585251335466 – Supplemental material for Personalized treatment decision algorithms for the clinical application of serum neurofilament light chain in multiple sclerosis: A modified Delphi Study Supplemental material, sj-pdf-5-msj-10.1177_13524585251335466 for Personalized treatment decision algorithms for the clinical application of serum neurofilament light chain in multiple sclerosis: A modified Delphi Study by Özgür Yaldizli, Pascal Benkert, Lutz Achtnichts, Amit Bar-Or, Viviane Bohner-Lang, Claire Bridel, Manuel Comabella, Oliver Findling, Giulio Disanto, Sebastian Finkener, Claudio Gobbi, Cristina Granziera, Marina Herwerth, Robert Hoepner, Dana Horakova, Nicole Kamber, Michael Khalil, Philipp Kunz, Patrice Lalive, Ralf Linker, Johannes Lorscheider, Stefanie Müller, Johanna Oechtering, Victoria Pettypool, Fredrik Piehl, Caroline Pot, Patrick Roth, Marie Théaudin, Mar Tintore, Carmen Tur, Denis Uffer, Marjolaine Uginet, Jochen Vehoff, Heinz Wiendl, Tjalf Ziemssen, Chiara Zecca, Anke Salmen, David Leppert, Tobias Derfuss, Ludwig Kappos, Lars G Hemkens, Perrine Janiaud and Jens Kuhle in Multiple Sclerosis Journal
