# Supplementary material for: Personalized treatment decision algorithms for the clinical application of serum neurofilament light chain in multiple sclerosis: A modified Delphi Study
Source: Mult Scler. 2025 Apr 28;31(8):932–43. doi: 10.1177/13524585251335466 (PMC12228887; doi:10.1177/13524585251335466)
Supplement: sj-docx-1-msj-10.1177_13524585251335466 – Supplemental material for Personalized treatment decision algorithms for the clinical application of serum neurofilament light chain in multiple sclerosis: A modified Delphi Study [file sj-docx-1-msj-10.1177_13524585251335466.docx]

**3Appendix – Excluded treatment decisions algorithms**

The following treatment decision algorithms were excluded after round 1

| **HORIZONTAL SWITCH WITH SHORTEN NATALIZUMAB INTERVAL** | | | |
| --- | --- | --- | --- |
| If your patient is currently receiving natalizumab every 6 weeks for at least 9 months and has high sNfL (>90thpercentile) consider to shorten the treatment interval to 4 weeks if your patient has: | | | |
| - **NEDA2** plus **MRI activity with at least 2** new/enlarging T2w lesions or contrast enhancing T1w lesions | 16% | 13% | 71% |
| - **NEDA2** plus **MRI activity with at least 1** new/enlarging T2w lesion or contrast enhancing T1w lesion | 13% | 13% | 74% |
| - **NEDA 3** | 31% | 38% | 31% |
|  | **Disagreed** | **Undecided** | **Agreed** |

| **HORIZONTAL SWITCH FROM B-CELL DEPLETING THERAPY** | | | |
| --- | --- | --- | --- |
| If your patient is currently receiving B-cell depleting therapy for at least 9 months and has high sNfL (>90th percentile) consider to switch to a different mode of action high efficacy DMT if your patient has: | | | |
| - **NEDA 3** | 50% | 30% | 20% |
|  | **Disagreed** | **Undecided** | **Agreed** |

| **DE-ESCALATION FROM NATALIZUMAB** | | | |
| --- | --- | --- | --- |
| If your patient is currently receiving natalizumab every 4 weeks for at least 2 years, has NEDA3 for the past 2 years and has normal sNfL (< 80th percentile) consider: | | | |
| - **Extending** the natalizumab **interval time to 6 weeks**. | 17% | 10% | 72% |
|  | **Disagreed** | **Undecided** | **Agreed** |

| **DE-ESCALATION FROM B-CELL DEPLETING THERAPY** | | | |
| --- | --- | --- | --- |
| If your patient is currently receiving B-cell depleting therapy for at least 5 years, has NEDA3 for the past 5 years and hasnormal sNfL (< 80th percentile) consider performing 6-monthly cMRI and 6-monthly sNfL measurement and de-escalate by: | | | |
| - Stopping DMT | 80% | 10% | 10% |
| - De-escalating to a medium efficacy DMT | 38% | 41% | 21% |
|  | **Disagreed** | **Undecided** | **Agreed** |

The following treatment decision algorithms were excluded during round 3 discussions with re-voting

| **DE-ESCALATION FROM B-CELL DEPLETING THERAPY** | | | |
| --- | --- | --- | --- |
| If your patient is currently receiving B-cell depleting therapy for at least 2 years, has NEDA3 for the past 2 years and has normal sNfL (< 80th percentile) jointly consider with your patient to perform 6-monthly cMRI and6-monthly sNfL measurement and de-escalate by: | | | |
| - Extending treatment interval to 12 months (Ocrevus, Rituximab) or 8 weeks (Kesimpta) as long asCD20 B cells are fully depleted (CD20 B cell count measurement frequency at the discretion of the physician) | 18% | 22% | 59% |
| - Extending treatment interval not to a fixed time period but as long as CD20 B cells are fully depleted(CD20 B cell count measurement frequency at the discretion of the physician) | 14% | 7% | 79% |
|  | **Disagreed** | **Undecided** | **Agreed** |

The following treatment decision algorithms were excluded following the vote during round 3

| **DE-ESCALATION FROM MEDIUM EFFICACY DMT** | | |
| --- | --- | --- |
| Algorithm: If your patient is >60 years old, currently receiving medium efficacy DMT for at least 5 years, has NEDA3 for the past 5 years, pre-treatment activity was low (e.g., less than 1 relapse per year) and has normal sNfL (<80th percentile) jointly consider with your patient to perform 6-monthly cMRI and 6-monthly sNfL measurement and: | | |
| - De-escalate from S1P | 65% | 26% |
| - De-escalation from fumarates | 35% | 48% |
|  | **Disagreed** | **Agreed** |
