# Supplementary material for: Personalized treatment decision algorithms for the clinical application of serum neurofilament light chain in multiple sclerosis: A modified Delphi Study
Source: Mult Scler. 2025 Apr 28;31(8):932–43. doi: 10.1177/13524585251335466 (PMC12228887; doi:10.1177/13524585251335466)
Supplement: sj-docx-3-msj-10.1177_13524585251335466 – Supplemental material for Personalized treatment decision algorithms for the clinical application of serum neurofilament light chain in multiple sclerosis: A modified Delphi Study [file sj-docx-3-msj-10.1177_13524585251335466.docx]

**Additional Methods: Voting rounds**

The first two rounds were online surveys using REDCap^1^. Only one person (PJ), overseeing the Delphi study, had access to REDCap, and was responsible for aggregating results and anonymizing comments. We used the REDCap built-in mailing tool to send out invitations and automatic reminders a week later. Each online round lasted for two weeks. Towards the end date personal reminders were sent by one researcher (ÖY) and the survey was left open for an additional 5 days.

Round 1 treatment decision algorithms were drafted by the core team based on their expertise and literature data. Between rounds the core team reviewed aggregated results and anonymized comments.

During round 1 and 2, the 9-point Likert scale was summarized as follows, 1 to 3 (bottom third) as disagreement, 4 to 6 as undecided and 7 to 9 (top third) as agreement, and a pre-defined threshold of consensus was set at ≥ 80% agreement.

For round 2, based on comments some algorithms were dropped and the remaining ones were rephrased or resubmitted as it, regardless if they had reached ≥ 80% agreement. All were accompanied with supportive evidence and explanations in responses to the comments raised in round 1.

During round 3, only treatment decision algorithms that had not reached a consensus of ≥ 80% agreement were discussed and amended through discussion during the in-person meeting when relevant. The in-person meeting was moderated by ÖY.

The final agreement thresholds were used: <50% excluded, 50% to 79% moderate consensus, 80% to 94% broad consensus, 95% to 99% strong consensus, and 100% full agreement.

1. Harris PA, Taylor R, Thielke R, et al. Research electronic data capture (REDCap)--a metadata-driven methodology and workflow process for providing translational research informatics support. *J Biomed Inform* 2009; 42: 377–381.
