# Supplementary material for: Personalized treatment decision algorithms for the clinical application of serum neurofilament light chain in multiple sclerosis: A modified Delphi Study
Source: Mult Scler. 2025 Apr 28;31(8):932–43. doi: 10.1177/13524585251335466 (PMC12228887; doi:10.1177/13524585251335466)
Supplement: sj-pdf-4-msj-10.1177_13524585251335466 – Supplemental material for Personalized treatment decision algorithms for the clinical application of serum neurofilament light chain in multiple sclerosis: A modified Delphi Study [file sj-pdf-4-msj-10.1177_13524585251335466.pdf]

MultiSCRIPT - Delphi Survey Round 1

Thank you for your participation. Please carefully read the introductions below.

Introduction

The following survey is the first round of the Delphi study for MultiSCRIPT within the Swiss MS Cohort.

The overall aim is to facilitate the implementation of sNfL information to guide escalation and de-escalation of disease modifying therapies (DMTs) in patients with relapsing-remitting multiple sclerosis (RRMS). We aim to provide a minimal set of treatment decision algorithms that experts have agreed upon for the most frequent clinical scenarios. Only statements that reach 100% consensus at the end of the three Delphi rounds will be retained.

Please carefully read the following:

The proposed treatment decision algorithms are not meant to cover all unique cases that physicians might encounter in the clinics. We acknowledge that treatment modifications related to pregnancies, treatment intolerances, comorbidities and other causes may not be captured here. The proposed algorithms are not binding and do not intend to supplant patients' and physicians' preferences. You will be asked to rate your agreement with treatment decision algorithms using a 9-Likert scale ranging from 1-Strongly disagree to 9-Strongly agree. Please provide a comment explaining any disagreement you might have.

DEFINITIONS:

Usual care arm: Consider escalation, if there is evidence of disease activity (clinically or MRI-activity) NEDA: no evidence of disease activity NEDA2: no relapse, no EDSS worsening NEDA3: no relapse, no EDSS worsening, no MRI activity EDSS worsening: defined as an increase of ≥1.5 points from an EDSS of 0, ≥1.0 point from an EDSS of 1.0-5.0 or ≥0.5 point from an EDSS ≥5.5 MRI activity: Any unequivocal new or enlarging T2w lesion (after adequate re-baselining) or contrast enhancement on T1w images on brain or spinal cord MRI according to the consensus between local neuroradiologist and treating neurologist Evidence of disease activity based on sNfL is defined using sNfL>90th percentile (single measurement)

Classification of DMTs:

Please classify the following approved MS drugs (currently used in the SMSC) into the following 3 groups with regard to their efficacy (i.e., overall effect on relapse rate and disease worsening):

1) Low efficacy DMT

2) Medium efficacy DMT

3) High efficacy DMT

	Low efficacy	Medium efficacy	High efficacy	I do not know
Alemtuzumab	○	○	○	○
Anti-B cell therapy	○	○	○	○
Cladribine	○	○	○	○
Fumarates	○	○	○	○
Glatiramer acetate	○	○	○	○
Interferon-beta	○	○	○	○
Natalizumab	○	○	○	○
S1P modulators	○	○	○	○
Teriflunomide	○	○	○	○

Please rate your agreement for the following statements (1-Strongly Disagree to 9-Strongly Agree)

N.B.: lesions are defined as any unequivocal new or enlarging T2w lesion (after adequate re-baselining) or contrast enhancement on T1w images on brain or spinal cord MRI according to the consensus between local neuroradiologist and treating neurologist

If your patient is currently untreated and has high sNfL (>90th percentile) consider to initiate DMT if your patient has.....

1) NEDA 3

______

Comments: ______

2) NEDA2 + MRI activity (at least 1 unequivocal new/enlarging T2w lesion or contrast enhancement)

______

Comments: ______

3) NEDA2 + MRI activity (at least 2 unequivocal new/enlarging T2w lesions or contrast enhancement)

______

Comments: ______

Please rate your agreement for the following statements (1-Strongly Disagree to 9-Strongly Agree)

N.B.: lesions are defined as any unequivocal new or enlarging T2w lesion (after adequate re-baselining) or contrast enhancement on T1w images on brain or spinal cord MRI according to the consensus between local neuroradiologist and treating neurologist

If your patient is currently receiving a low efficacy DMT for at least 9 months and has high sNfL (>90th percentile) consider to escalate to medium or high efficacy DMT if your patient has.....

1) NEDA 3

______

Comments: ______

2) NEDA2 + MRI activity (at least 1 unequivocal new/enlarging T2w lesion or contrast enhancement)

______

Comments: ______

3) NEDA2 + MRI activity (at least 2 unequivocal new/enlarging T2w lesions or contrast enhancement)

______

Comments: ______

Please rate your agreement for the following statements (1-Strongly Disagree to 9-Strongly Agree)

N.B.: lesions are defined as any unequivocal new or enlarging T2w lesion (after adequate re-baselining) or contrast enhancement on T1w images on brain or spinal cord MRI according to the consensus between local neuroradiologist and treating neurologist

If your patient is currently receiving a medium efficacy DMT for at least 9 months and has high sNfL (>90th percentile) consider to escalate to high efficacy DMT if your patient has.....

1) NEDA 3

______

Comments: ______

2) NEDA2 + MRI activity (at least 1 unequivocal new/enlarging T2w lesion or contrast enhancement)

______

Comments: ______

3) NEDA2 + MRI activity (at least 2 unequivocal new/enlarging T2w lesions or contrast enhancement)

______

Comments: ______

Please rate your agreement for the following statements (1-Strongly Disagree to 9-Strongly Agree)

N.B.: lesions are defined as any unequivocal new or enlarging T2w lesion (after adequate re-baselining) or contrast enhancement on T1w images on brain or spinal cord MRI according to the consensus between local neuroradiologist and treating neurologist

If your patient is currently receiving natalizumab every 6 weeks for at least 9 months and has high sNfL (>90th percentile) consider to shorten the treatment interval to 4 weeks if your patient has.....

1) NEDA 3

______

Comments: ______

2) NEDA2 + MRI activity (at least 1 unequivocal new/enlarging T2w lesion or contrast enhancement)

______

Comments: ______

3) NEDA2 + MRI activity (at least 2 unequivocal new/enlarging T2w lesions or contrast enhancement)

______

Comments: ______

Please rate your agreement for the following statements (1-Strongly Disagree to 9-Strongly Agree)

N.B.: lesions are defined as any unequivocal new or enlarging T2w lesion (after adequate re-baselining) or contrast enhancement on T1w images on brain or spinal cord MRI according to the consensus between local neuroradiologist and treating neurologist

If your patient is currently receiving natalizumab every 4 weeks for at least 9 months and has high sNfL (>90th percentile) consider to switch to a different mode of action high effficacy DMT if your patient has.....

1) NEDA 3

______

Comments: ______

2) NEDA2 + MRI activity (at least 1 unequivocal new/enlarging T2w lesion or contrast enhancement)

______

Comments: ______

3) NEDA2 + MRI activity (at least 2 unequivocal new/enlarging T2w lesions or contrast enhancement)

______

Comments: ______

Please rate your agreement for the following statements (1-Strongly Disagree to 9-Strongly Agree)

N.B.: lesions are defined as any unequivocal new or enlarging T2w lesion (after adequate re-baselining) or contrast enhancement on T1w images on brain or spinal cord MRI according to the consensus between local neuroradiologist and treating neurologist

If your patient is currently receiving anti-B cell therapy for at least 9 months and has high sNfL (>90th percentile) consider to switch to a different mode of action high efficacy DMT if your patient has.....

1) NEDA 3

______

Comments: ______

2) NEDA2 + MRI activity (at least 1 unequivocal new/enlarging T2w lesion or contrast enhancement)

______

Comments: ______

3) NEDA2 + MRI activity (at least 2 unequivocal new/enlarging T2w lesions or contrast enhancement)

______

Comments: ______

Please rate your agreement for the following statements (1-Strongly Disagree to 9-Strongly Agree)

N.B.: lesions are defined as any unequivocal new or enlarging T2w lesion (after adequate re-baselining) or contrast enhancement on T1w images on brain or spinal cord MRI according to the consensus between local neuroradiologist and treating neurologist

If your patient is currently receiving Cladribine for at least 9 months and has high sNfL (>90th percentile) consider escalating DMT if your patient has .....

1) NEDA 3

______

Comments: ______

2) NEDA2 + MRI activity (at least 1 unequivocal new/enlarging T2w lesion or contrast enhancement)

______

Comments: ______

3) NEDA2 + MRI activity (at least 2 unequivocal new/enlarging T2w lesions or contrast enhancement)

______

Comments: ______

Please rate your agreement for the following statements (1-Strongly Disagree to 9-Strongly Agree)

If your patient is currently receiving natalizumab every 4 weeks for at least 2 years, has NEDA3 for the past 2 years and has normal sNfL (< 80th percentile) consider extending the natalizumab interval time to 6 weeks.

______

Comments: ______

Please rate your agreement for the following statements (1-Strongly Disagree to 9-Strongly Agree)

If your patient is currently receiving anti-B cell therapy for at least 5 years, has NEDA3 for the past 5 years and has normal sNfL (< 80th percentile) consider performing 6-monthly cMRI and 6-monthly sNfL measurement and de-escalate by

1) Stopping DMT

______

Comments: ______

2) De-escalating to a medium efficacy DMT

______

Comments: ______

3) Extending treatment interval to 12 months (Ocrevus, Rituximab) or 8 weeks (Kesimpta)

______

Comments: ______

4) Extending treatment interval as long as CD19 B cell count is fully depleted (6 monthly CD19 B cell count measurements)

______

Comments: ______

Please rate your agreement for the following statements (1-Strongly Disagree to 9-Strongly Agree)

If your patient is currently receiving medium efficacy DMT for at least 5 years, has NEDA3 for the past 5 years and has normal sNfL (< 80th percentile) consider performing 6-monthly cMRI and 6-monthly sNfL measurement and....

1) De-escalating to low efficacy DMT

______

Comments: ______

2) Stopping DMT

Comments: ______

Please rate your agreement for the following statements (1-Strongly Disagree to 9-Strongly Agree)

If your patient is currently receiving low efficacy DMT and NEDA3 for the past 5 years, and has normal sNfL (< 80th percentile) consider performing 6-monthly cMRI and 6-monthly sNfL measurement and stopping DMT

______

Comments: ______
