# Supplementary material for: Personalized treatment decision algorithms for the clinical application of serum neurofilament light chain in multiple sclerosis: A modified Delphi Study
Source: Mult Scler. 2025 Apr 28;31(8):932–43. doi: 10.1177/13524585251335466 (PMC12228887; doi:10.1177/13524585251335466)
Supplement: sj-pdf-5-msj-10.1177_13524585251335466 – Supplemental material for Personalized treatment decision algorithms for the clinical application of serum neurofilament light chain in multiple sclerosis: A modified Delphi Study [file sj-pdf-5-msj-10.1177_13524585251335466.pdf]

MultiSCRIPT - Delphi Survey Round 2

Thank you for you participation. Please carefully read the introduction below.

Introduction

The following survey is the 2nd round of the Delphi for MultiSCRIPT within the Swiss MS Cohort (SMSC). Based on your feedback on the 1st round, we here provide more information on the rationale of the proposed treatment decision algorithms with respective references.

Overview of the 1st round:

Out of the 32 email invitations sent, we collected 31 completed surveys. Responders included 18 physicians participating in the SMSC, 10 international experts and 3 persons with MS (as consultants). In total, 29 treatment decision algorithms on 11 topics were submitted for your assessment: 9 have reached an 80%-consensus (that means they received ratings of 7 to 9 on the 9-point Likert scale), and one was rated 1 to 3 by >80%.

Comments from round 1:

In the following paragraphs, we are addressing some of the most common comments made during the 1st round. To see all comments please refer to the report available here.

One common comment was the necessity of first excluding other causes of high sNfL values. Similarly, survey participants frequently raised the importance of taking into account other factors such as age, disease duration, MRI lesion count, etc.

We agree with these comments and would like to emphasize that, of course, the treatment decision algorithms should only be applied if other potential causes explaining high sNfL have been excluded. Furthermore, factors such as comorbidities, neutralizing antibodies, among others, which may lead to suboptimal treatment effect, need to be factored in. The suggested algorithms assume that such special conditions do NOT apply. We do acknowledge that MS is a heterogeneous and complex disease. With the Delphi study, we aim to provide a minimal set of treatment decision algorithms that experts have agreed upon for the most frequent clinical scenarios.

Moreover, patients' preference was frequently highlighted along with the concern in the de-escalation questions, on the risk of changing a treatment that works and has achieved NEDA3. Please note, the treatment decision algorithms through the Delphi study are merely general recommendations to be considered jointly with your patient and are non-binding. Patients and treating physician can always overrule the recommendations made here in the Delphi process.

Many comments also referred to MRI. MRI activity is defined as contrast enhancing lesion or the unequivocal presence of a new/enlarging T2w lesion. We consider in this study a new/enlarging T2w lesion as evidence of MRI activity only if it occurs after a comparable adequately re-baselined scan and is based on the consensus of neuroradiologist and neurologist.

Definitions

You will now be asked to assess the treatment decision algorithms for Round 2.

The overall aim of this study is to assess the value of implementing sNfL information to guide escalation and de-escalation of disease modifying therapies (DMTs) in patients with relapsing-remitting multiple sclerosis (RRMS). The proposed algorithms aim to provide a minimal set of treatment decision algorithms that experts have agreed upon for the most frequent clinical scenarios. Only statements that reach 100% consensus (defined as ratings 7-9) at the end of the three Delphi rounds will be retained.

You will be asked to rate your agreement with treatment decision algorithms using a 9-Likert scale ranging from 1-Strongly disagree to 9-Strongly agree.

Please provide a comment explaining any disagreement you might have.

DEFINITIONS: (please notes the edits compared to round 1 are highlighted in blue)

Usual care arm: Consider escalation, if there is evidence of disease activity (clinically or MRI-activity)

NEDA: no evidence of disease activity

NEDA2: no relapse, no EDSS worsening

NEDA3: no relapse, no EDSS worsening, no MRI activity

EDSS worsening: defined as an increase of ≥1.5 points from an EDSS of 0, ≥1.0 point from an EDSS of 1.0-5.0 or ≥0.5 point from an EDSS ≥5.5

MRI activity: Any unequivocal new or enlarging T2w lesion (after adequate re-baselining) or contrast enhancement on T1w images on brain or spinal cord MRI according to the consensus between local neuroradiologist and treating neurologist.

Evidence of disease activity based on sNfL is defined using sNfL>90th percentile (Benkert et al. Lancet Neurology 2022) and once other potential causes of high sNfL have been excluded (e.g. trauma, stroke, relevant sports-related head injury, at least medium severe renal failure (GFR < 60 mL/min/1.73 m2), suboptimally treated diabetes mellitus or any other concomitant disease that may lead to relevant neuroaxonal damage)

Please rate your agreement (1-Strongly disagree to 9-Strongly agree) for the following grouping of MS drugs:

1) Low efficacy: Glatiramer acetate - Interferon-beta - Teriflunomide

______

Comments: ______

2) Medium efficacy: Fumarates - S1P modulators - Cladribine

______

Comments: ______

3) High efficacy: Alemtuzumab - Anti B cell therapy - Natalizumab

______

Comments: ______

Results round 1:

______

Comments:

In the 1st round, broad consensus was achieved for all compounds except for teriflunomide, fumarates and cladribine. Please take into account that grouping in 3 categories does not deny differences of compounds listed within each of the categories. It does only reflect that differences within each group are less pronounced than between the groups. Teriflunomide, fumarates and cladribine were here assigned to groups based on the majority of ratings to explore if a higher consent rate can be achieved.

Please rate your agreement for the following statements (1-Strongly Disagree to 9-Strongly Agree)

If your patient is currently untreated and has high sNfL (>90th percentile) jointly consider with your patient to initiate DMT if your patient has.....

1) NEDA 3

______

Comments: ______

2) NEDA2 + MRI activity (at least 1 unequivocal new/enlarging T2w lesion or contrast enhancement)

______

Comments: ______

3) NEDA2 + MRI activity (at least 2 unequivocal new/enlarging T2w lesions or contrast enhancement)

______

Comments: ______

Results round 1:

______

Comments:

We have already achieved almost consensus for initiation of DMT in case of MRI activity and high sNfL. For the scenario of isolated high sNfL and NEDA3, we have not achieved agreement. Please note that the RRMS patients in this question are untreated. In general, the European guidelines already recommend to initiate a DMT in RRMS (Montalban et al. MSJ 2018). There are 58 patients in the SMSC who are untreated. The question here is whether or not we want to recommend to initiate a DMT in case of high sNfL, which indicates increased neuro-axonal damage based on its specificity for neuronal injury, captures also affection of the spinal cord and has shown to be more sensitive than conventional (brain) MRI (Benkert et al., Lancet Neurology 2022). We will use a single measurement of sNfL: MultiSCRIPT is a pragmatic trial, i.e. that should not change routine care, and is embedded in the framework of the SMSC with 6 monthly visits.

Escalation from low to medium or high DMT:

Please rate your agreement for the following statements (1-Strongly Disagree to 9-Strongly Agree)

If your patient is currently receiving a low efficacy DMT for at least 9 months and has high sNfL (>90th percentile) jointly consider with your patient to escalate to medium or high efficacy DMT if your patient has.....

1) NEDA 3

______

Comments: ______

2) NEDA2 + MRI activity (at least 1 unequivocal new/enlarging T2w lesion or contrast enhancement)

______

Comments: ______

3) NEDA2 + MRI activity (at least 2 unequivocal new/enlarging T2w lesions or contrast enhancement)

______

Comments: ______

Escalation from medium to high DMT

Please rate your agreement for the following statements (1-Strongly Disagree to 9-Strongly Agree)

If your patient is currently receiving a medium efficacy DMT for at least 9 months and has high sNfL (>90th percentile) jointly consider with your patient to escalate to high efficacy DMT if your patient has.....

1) NEDA 3

______

Comments: ______

2) NEDA2 + MRI activity (at least 1 unequivocal new/enlarging T2w lesion or contrast enhancement)

______

Comments: ______

3) NEDA2 + MRI activity (at least 2 unequivocal new/enlarging T2w lesions or contrast enhancement)

______

Comments: ______

Results round 1:

Escalation from low to medium or high DMTs

______

Escalation from medium to high DMTs

______

Comments:

The two questions above refer to the situation that a patient with RRMS is treated with a low or medium DMT.

Do we want to recommend to these patients a higher efficacy drug if patient is NEDA3 but sNfL is increased? Results from previous studies (Benkert et al. Lancet Neurology 2022 and several others) have shown that an increased sNfL is independently of other clinical or MRI measures associated with MS disease activity in the following year (Figure 1; i.e., also in NEDA3 patients indicating that sNfL is able to capture neuro-axonal injury at higher sensitivity than conventional MRI/clinical assessment which is important given also the limited prognostic capacity of NEDA3 status for future progression; i.e., ongoing neuro-axonal damage that is captured by sNfL). Many studies over the past 5 years (also in controlled settings; Kuhle et al. Neurology 2019, Kuhle et al. MSJ 2022, Benkert et al. Lancet Neurology 2022) showed that patients treated with higher efficacy drugs lead to lower sNfL values compared to untreated patients or patients with lower efficacy drugs (Figure 2). This is the rationale for the question to escalate from low or medium efficacy DMT based on the added sNfL information. Please note, in contrast to new/enlarging T2w lesions on MRI that is a proxy for disease activity since last MRI, sNfL is a measure that is associated with disease activity more closely to the time point of the assessment. Previous studies (e.g. Benkert et al. Lancet Neurology 2022) have shown that sNfL can be increased in NEDA3 patients suggesting that sNfL and MRI are at least partly independent from each other. Moreover, sNfL is a systemic measure easy to assess. In contrast to sNfL, it is unrealistic to obtain 6 monthly cranial and spinal cord MRI over a long period of time as a treatment monitoring tool in apparently clinically stable MS.

Several studies have shown that patients with high sNfL under an established treatment have a worse disease course compared to patients who show normal NfL levels under this treatment (Figure 3; Benkert et al. Lancet Neurology 2022; Cohen JA et al. Ther Advan Neurol Dis 2019; Kuhle et al. data presented at ECTRIMS 2019; Bar-Or et al. data presented at ECTRIMS 2022)

______

Figure 1: This multivariable analysis from the SMSC shows that increased sNfL levels are associated with an increased risk of clinical or MRI disease activity in the following year (n=1313 MS patients; 23% increased risk per Z score unit change in sNfL) independent of EDSS worsening, relapse rate in the previous year and MRI activity (Benkert et al. Lancet Neurology 2022).

______

Figure 2: This data from the SMSC shows that sNfL is decreasing in patients on high efficacy DMT in contrast to untreated patients or patients on low efficacy DMT (Benkert et al. Lancet Neurology 2022).

______

______

Figure 3: Data from fingolimod treated RRMS patients in the LONGTERMS study. High sNfL at month (M)6 and/or month 12 under fingolimod treatment doubled the risk of future disability progression and time to EDSS 4 (Cohen JA et al. Ther Advan Neurol Dis 2019; Kuhle J et al. data presented at ECTRIMS 2019) indicating suboptimal treatment response.

Please rate your agreement for the following statements (1-Strongly Disagree to 9-Strongly Agree)

If your patient is currently receiving natalizumab for at least 9 months and has high sNfL (>90thpercentile) jointly consider with your patient to switch to a different mode of action high efficacy DMT if your patient has.....

1) NEDA 3

______

Comments: ______

2) NEDA2 + MRI activity (at least 1 unequivocal new/enlarging T2w lesion or contrast enhancement)

______

Comments: ______

3) NEDA2 + MRI activity (at least 2 unequivocal new/enlarging T2w lesions or contrast enhancement)

______

Comments: ______

Results round 1:

______

Comments:

Most of the survey participants in this Delphi process would switch from natalizumab to a different mode of action high efficacy DMT (e.g. anti B cell therapy), if patient has high sNfL and MRI activity.

The critical question here is whether or not a high sNfL in a NEDA3 patient would be also sufficient to recommend to switch natalizumab to e.g. anti-B cell therapy. The rationale behind this question is that high sNfL in treated MS patients is a signal of suboptimal treatment response (see previous page Figure 4). Even if there is e.g. probably no significant difference in the efficacy of natalizumab and anti-B cell therapy, on an individual basis, a high efficacy DMT with a different mode of action might control here disease activity more efficiently than natalizumab.

Please rate your agreement for the following statements (1-Strongly Disagree to 9-Strongly Agree)

If your patient is currently receiving anti-B cell therapy for at least 9 months and has high sNfL (>90th percentile) jointly consider with your patient to switch to a different mode of action high efficacy DMT if your patient has.....

1) NEDA2 + MRI activity (at least 1 unequivocal new/enlarging T2w lesion or contrast enhancement)

______

Comments: ______

2) NEDA2 + MRI activity (at least 2 unequivocal new/enlarging T2w lesions or contrast enhancement)

______

Comments: ______

3) No MRI activity but confirmed EDSS worsening or relapse

______

Comments: ______

4) MRI activity and confirmed EDSS worsening or relapse

______

Comments: ______

Results round 1:

______

Comments:

Most of the raters in this Delphi process would not switch from an anti-B cell therapy if there is high sNfL in clinically and MRI stable RRMS patients (NEDA3). That is why we deleted this option in the 2nd round.

The question now is whether you would consider switching to anti-B cell therapy if there is MRI activity plus high sNfL. This is a relevant question. In the SMSC, 15% of patients on anti-B cell therapy for at least 9 months have high sNfL; 23 patients on anti-B cell therapy switched to a DMT with a different mode of action (i.e., the question is if this may be a viable route e.g., switch to natalizumab in JCV serology negative patients). A rationale for a switch are data from the OPERA studies (same results in the ORATORIO trial): RRMS patients on ocrelizumab with high sNfL at week 48 after treatment start had a significantly higher chance for futute disability worsening up to 9 years after starting the DMT (Figure 4; Bar-Or A et al. data presented at ECTRIMS 2022)

Figure 4: In RRMS patients on ocrelizumab, high sNfL at week 48 was associated with confirmed disability worsening (Bar-Or A et al. data presented at ECTRIMS 2022)

Please rate your agreement for the following statements (1-Strongly Disagree to 9-Strongly Agree)

If your patient is at least 6 months after receiving the second cycle cladribine and has high sNfL (>90th percentile) jointly consider with your patient to escalate/switch DMT if your patient has .....

1) NEDA 3

Comments:

2) NEDA2 + MRI activity (at least 1 unequivocal new/enlarging T2w lesion or contrast enhancement)

Comments:

3) NEDA2 + MRI activity (at least 2 unequivocal new/enlarging T2w lesions or contrast enhancement)

Comments:

Results round 1:

Comments:

It was not clear in this question which cycle of cladribine was meant. We have now clarified this question and would like to ask you again whether you would switch/escalate from Cladribine in case of high sNfL only or only in case of MRI activity plus high sNfL.

Please rate your agreement for the following statements (1-Strongly Disagree to 9-Strongly Agree)

If your patient is currently receiving anti-B cell therapy for at least 2 years, has NEDA3 for the past 2 years and has normal sNfL (< 80th percentile) jointly consider with your patient to perform 6-monthly cMRI and 6-monthly sNfL measurement and de-escalate by.....

1) Extending treatment interval to 12 months (Ocrevus, Rituximab) or 8 weeks (Kesimpta)

Comments:

2) Extending treatment interval to 12 months (Ocrevus, Rituximab) or 8 weeks (Kesimpta) as long as CD20 B cells are fully depleted (CD20 B cell count measurement frequency at the discretion of the physician)

Comments:

3) Extending treatment interval not to a fixed time period but as long as CD20 B cells are fully depleted (CD20 B cell count measurement frequency at the discretion of the physician)

Comments:

Results round 1:

Comments:

The strongest agreement was reached for extending the dose interval of anti B cell therapy in case of NEDA3 and low sNfL. As the effects of anti B cell therapy on MRI measures are already seen after 6 months (Hauser SL et al. NEJM 2017), we would like to ask you in this round whether you would also consider to extend intervals of anti B-cell therapy after 2 years of stability (NEDA3 and low sNfL). Please note, for monitoring reason, all these patients will have 6 monthly visits including MRI and sNfL. In case of MRI activity or increase in sNfL anti-B cell therapy can be re-initiated.

The other question is whether you think it is important to extend the treatment interval individually based on peripheral CD20 counts or independent of the CD20 count from 6 to 12 months.

Please note, that patients who de-escalate cannot do better in terms of relapse rate and EDSS worsening compared

to patients continuing anti-B cell therapy but de-escalation might increase the quality of life in these patients due to fewer infusions, less risk for side effects/risks (also longterm) and reduce costs.

Please rate your agreement for the following statements (1-Strongly Disagree to 9-Strongly Agree)

If your patient is >60 years old, currently receiving medium efficacy DMT for at least 5 years, has NEDA3 for the past 5 years, pre-treatment activity was low (e.g., less than 1 relapse per year) and has normal sNfL (< 80th percentile) jointly consider with your patient to perform 6-monthly cMRI and 6-monthly sNfL measurement and ....

1) De-escalate to low efficacy DMT

______

Comments: ______

2) Stop DMT

______

Comments: ______

Results round 1:

______

Comments:

Most participants would neither de-escalate nor stop DMT in a patient on medium efficacy DMT in case of NEDA3.

In answer to your comments, we have added here the age limit of 60 years of age. Currently in the SMSC, there are 45 patients with RRMS over 60 years of age treated with medium efficacy DMTs for at least 5 years (median 8.4 years).

All, except 2, have been relapse-free in the past 5 years. We believe these patients may be safely de-escalated in the intensive treatment monitoring arm of this study. Again, please note that these patients have close monitoring with 6-monthly visits, cMRI and spinal MRI and sNfL. Please also note, that medium efficacy drugs are oral DMTs. Most of the patients will probably not de-escalate from an oral DMT to an injectable. However, if we reach consensus with teriflunomide being in the low efficacy DMT group, older RRMS patients on a medium efficacy drug might de-escalate to teriflunomide as an alternative e.g. to minimize the risk of opportunistic infections

---

Please rate your agreement for the following statements (1-Strongly Disagree to 9-Strongly Agree)

If your patient is >60 years old, currently receiving low efficacy DMT for at least 5 years, has NEDA3 for the past 5 years and has normal sNfL (< 80th percentile) jointly consider with your patient to perform 6-monthly cMRI and 6-monthly sNfL measurement and...

1) Stop DMT

______

Comments: ______

Results round 1:

______

Comments:

Similar to the last question, here, most of the raters would not stop DMT in a patient on low efficacy DMT who are NEDA3 for 5 years. Based on your comments, we have added here again the age limit of 60 years.

Please note, most of the low efficacy DMTs are injectables. Currently, there are 16 patients with RRMS in the SMSC, over 60 years of age and treated with low efficacy DMTs for at least >5 years (median 7.8 years). All except 1 have been relapse-free in the past 5 years and could benefit from stopping DMT with increase in quality of life without disease activity.
